# Determinants of Highly Active Antiretroviral Therapy Duration in HIV-1-Infected Children and Adolescents in Madrid, Spain, from 1996 to 2012

**DOI:** 10.1371/journal.pone.0096307

**Published:** 2014-05-01

**Authors:** Claudia Palladino, Verónica Briz, José María Bellón, Francisco J. Climent, Santiago J. de Ory, María José Mellado, María Luisa Navarro, José T. Ramos, Nuno Taveira, María Isabel de José, María Ángeles Muñoz-Fernández, on the behalf of the CoRISpeS-Madrid Cohort Working Group

**Affiliations:** 1 Instituto de Investigação do Medicamento (iMed. ULisboa), Faculty of Pharmacy, The University of Lisbon, Lisbon, Portugal; 2 Hospital General Universitario “Gregorio Marañón” and Instituto de Investigación Sanitaria “Gregorio Marañón”, Madrid, Spain. Networking Research Center on Bioengineering, Biomaterials and Nanomedicine (CIBER-BBN), Madrid, Spain; 3 Unidad de Investigación, Fundación para la Investigación Biomédica, Hospital General Universitario “Gregorio Marañón”, Madrid, Spain; 4 Servicio Infecciosas Infantil, Hospital Universitario “La Paz”, Madrid, Spain; 5 Servicio de Pediatría, Hospital Universitario “Carlos III”, Madrid, Spain; 6 Servicio de Pediatría, Hospital General Universitario “Gregorio Marañón”, Madrid, Spain; 7 Servicio de Pediatría, Hospital Universitario de Getafe, Madrid, Spain; 8 Center of Interdisciplinary Investigation Egas Moniz (CiiEM), Institute of Health Sciences Egas Moniz, Caparica, Portugal; University of Texas Health Science Center San Antonio Texas, United States of America

## Abstract

**Objectives:**

To investigate the duration of sequential HAART regimens and predictors of first-line regimen discontinuation among HIV-1 vertically infected children and adolescents.

**Design:**

Multicentre survey of antiretroviral-naïve patients enrolled in the HIV-Paediatric Cohor,t CoRISpeS-Madrid Cohort, Spain.

**Methods:**

Patients with a follow-up of ≥1 month spent on HAART, with available baseline CD4 count and HIV-viral load (VL) were included. Time spent on sequential HAART regimens was estimated and multivariable regression was used to identify predictors of time to first-line regimen discontinuation.

**Results:**

104 patients were followed for a median 8 years after starting HAART among 1996–2012; baseline %CD4 was 21.5 (12.3–34.0)and viral load was 5.1 (4.6–5.6) log_10_ copies/mL. Patients received a mean of 1.9 regimens. Median time on first-line HAART (n = 104) was 64.5 months; second HAART (n = 56) 69.8 months; and third HAART (n = 21) 66.5 months. Eleven (11%) patients were lost to follow-up while on first-line HAART and 54% discontinued (cumulative incidence of 16% and 38% by 1 and 3-year, respectively). The main predictor of first-line regimen discontinuation was suboptimal adherence to antiretrovirals (AHR: 2.60; 95% CI: 1.44–4.70).

**Conclusions:**

Adherence to therapy was the main determinant of the duration of the first-line HAART regimen in children. It is important to identify patients at high risk for non-adherence, such as very young children and adolescents, in provide special care and support to those patients.

## Introduction

The introduction of highly active antiretroviral therapy (HAART) to medical care for vertically HIV-1-infected children has increased life expectancy and resulted in a substantial decline in AIDS incidence [1]–[8]. However, the paucity of antiretroviral choices and the survival to adolescence and adulthood of HIV-1-infected children pose main concerns about HAART effectiveness. Worldwide, there is a growing cohort of adolescents who are long-term survivors of vertically-acquired HIV-1 infection, who are likely to modifying the shape of the HIV epidemic in the next future. Being these patients heavily HAART-experienced and facing developmental problems, these patients are characterised by unique features which may hamper the impact of HAART. In particular, the duration of first-line HAART is critical in the context of the lifelong treatment of these patients. Several factors that have been shown to limit the success of HAART in children are the patient characteristics [9], lack of adherence [10], [11], lack of appropriate dosing [12], [13], drug tolerability and toxicity [14]–[16], and viral resistance [17]. In Spain, a free access to HAART has been universal since 1996, leading to a dramatic decrease of AIDS and mortality [18]. Nonetheless, prevalence in Spain is still one of the highest HIV-1 epidemic in Western Europe [19]. Within Spain, the *Comunidad Autónoma de Madrid* accounted for the 26% of the AIDS cases associated to HIV-1 vertical transmission, most of them now, older children [20].

In this study, we evaluate the determinants of first-line HAART discontinuation in a large cohort of HIV-1-infected paediatric patients from Madrid, Spain. The findings of this study provide valuable information for the management of paediatric HIV-1-infection.

## Paediatric Patients and Methods

### Ethics Statement

The study was conducted according to the Declaration of Helsinki and was approved by the Ethical Committees of each participating hospital of the HIV Paediatric Cohort (CoRISpeS-Madrid Cohort) working group. Written informed consent was obtained from all parents or tutors.

### The HIV Paediatric Cohort of the Comunidad Autónoma de Madrid

The HIV Paediatric Cohort of the *Comunidad Autónoma de Madrid* (CoRISpeS-Madrid Cohort) was established in 1995 as an open cohort of all HIV-1 vertically-infected children identified in a multicentre network of referral paediatric hospitals based in the *Comunidad Autónoma de Madrid* (see Annex). HIV-1 vertical transmission was assumed to have occurred on the date of birth [21]. Children were enrolled from 1982 (birth date of the first case of HIV-1 vertical transmission in Madrid) to the present and were monitored through active follow-up every 3–6 months according to published guidelines [22]. In this cohort, children infected before 1995 were enrolled retrospectively while children infected after 1995 were enrolled prospectively. Complete ascertainment of all records was carefully sought and cross-check with the National AIDS Reports was performed. The cohort included 491 HIV-1 infected children and adolescents as of December 2012.

The present survey was conducted according to the Declaration of Helsinki with the approval of the Institutional Ethics Committee. Patients were selected retrospectively for this survey and were eligible if they had no history of antiretroviral treatment when they initiated their first HAART regimen. The other eligible criteria were: a follow-up of ≥1 month spent on HAART; available baseline CD4 count and HIV-1 RNA viral load (VL). The study period for inclusion of patients was from 1996 to 2012. Individuals were followed from the date of HAART initiation until the date of death, or date of last visit in case of lost to follow-up, or December 31, 2012 (administrative censoring date), which ever occurred first. VL was measured by Amplicor Monitor assay (Roche, Brandenburg, NJ) with a detection limit 50 copies/mL. Several biological samples were provided by the HIV HGM BioBank [23].

Demographics, clinical, immunological and virological data and HAART use were gathered retrospectively. Clinical classification of AIDS-defining events was based on international guidelines [24], [25]. All patients included in the study were eligible for HAART initiation (i.e., any combination of ≥3 antiretrovirals, excluding antiretroviral prophylaxis) [26]. A HAART regimen was counted when it had been administered for at least 30 days (to ensure that patients were genuinely taking their antiretrovirals). A regimen discontinuation was defined as changing ≥2 antiretrovirals simultaneously while continuing on HAART or an interruption of >30 days. Causes of initial HAART discontinuation were extracted from medical charts, confirmed with paediatricians, and classified as poor adherence, treatment failure, toxicity, simplification, planned treatment interruption, decision of patient/parents/tutor, social/family problem, death. Baseline laboratory values were measured at the time of first regimen initiation (range: −6 months; +14 days). Laboratory values at regimen discontinuation were determined using the same window around the discontinuation date. Calendar period for HAART initiation (divided into 1996–2001 and 2002–2012) was categorised *a priori*, because approval of lopinavir/ritonavir could have led to change in treatment management [27]–[29]. Clinicians monitored adherence by pill count and interviewing parents or tutors at each visit during follow-up. Adherence was summarized as a single percentage for each HAART regimen for each patient and categorized as poor (<70%), intermediate (70–90%), good (91%–99%), perfect (100%). Lost to follow-up was defined as no communication between the patient and the clinic staff resulting in missing at least 3 visits.

### Statistical Analysis

Demographic and baseline characteristics and antiretroviral therapy use are presented using descriptive statistics. The primary endpoint was the time spent on sequential regimens and was estimated by Kaplan-Meier analysis with exact 95% confidence intervals (CI); log-rank test was used to compare survival curves. Time was calculated from the date of HAART initiation until the date of HAART change, or last visit in case of lost to follow-up, or December 31, 2012 (administrative censoring date), whichever occurred first. Univariate proportional hazards regression analyses were performed to identify factors associated with first-line regimen discontinuation, and multivariable proportional hazards regression analysis was performed that included all factors for which the results of univariate analysis were statistically significant (P<0.05) or close to the margin of statistical significance. The variables examined included demographic characteristics, social indicators, baseline laboratory values, HIV-1 subtype, clinical status, provision of antiretroviral prophylaxis to the paediatric patients and calendar period for HAART initiation.

To ensure that outcome events could be correctly attributable to the type of regimen, when a substitution of a single drug caused the change from a protease inhibitor (PI)-based to a non-nucleoside reverse transcriptase (NNRTI)-base regimen or vice versa, the type of regimen was re-categorised on the basis of the longest regimen for each patient. Analyses were performed by using SPSS (v.19; Chicago, IL) and 2-tailed P-values <0.05 were considered statistically significant.

## Results

Of 124 antiretroviral-naïve children who started HAART, 104 were eligible. They initiated HAART between 1997–2012 and had a median time of observation of 101.0 (51.9–139.8) months. At baseline, the majority of children (56/104; 53.8%) had VL >100.000 copies/mL and %CD4<25 (65/104; 62.5%). Other baseline characteristics are listed in Table S1 in File S1. Remarkably, a tendency to a better adherence among median aged-children (2–12 years old) than among very young children (≤2 years old) and adolescents (>12 years old) was observed, with P-value close to the margin of statistical significance (P = 0.063). Overall, a rapid and sustained increase in median %CD4 and concurrent decrease in %CD8 and VL was observed during follow-up (Figure S1A in File S1). A comparative analysis among patients included (n = 104) and not included (n = 20) in the survey showed that the two groups were similar in the following characteristics: demographics, clinical status, breastfeeding, maternal transmission category and age at HAART initiation (Table S2 in File S1).

### Antiretroviral Therapy Duration

**Figure 1:**
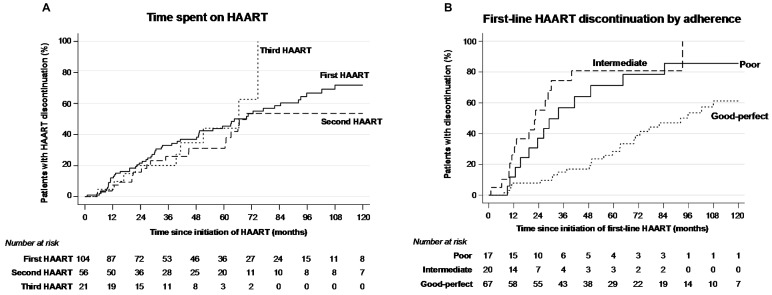
Kaplan–Meier survival curves for time spent on first vs. second vs. third HAART (A) and for time spent on first HAART by adherence to antiretrovirals (B).

Overall, the mean number of HAART regimens used was 1.9. Eleven (10.6%) children were lost to follow-up while on first-line HAART, 56/104 (53.8%) discontinued their initial regimen, one (1.0%) died of dideoxynucleoside-induced severe lactic acidosis and 36/104 (34.6%) remained under their first-line therapy. Of those, 10/36 (27.8%) had a single-drug substitution within the same class and 2/36 (5.6%) had a class change. Comparison with initial HAART regimen to successive regimens revealed that fewer salvage regimens based on a single PI and more with a boosted PI or four drugs were used (Table S1 in File S1). After 2007, when nelfinavir was withdrawn from the European market, there was a considerable increase in the use of lopinavir/ritonavir (Figure S1B in File S1). Median time on first-line regimen was 64.5 months (47.5–81.5), similar to time spent on second and third-line regimens (Table S1 in File S1; Figure 1A). Treatment failure was the main cause of first-line therapy discontinuation (19/57, 33.3%), followed by poor adherence (12/57, 21.1%) and simplification (10/57, 17.5%) (Table S3 in File S1). Time spent receiving first-line HAART was shorter among individuals with poor adherence to antiretrovirals than among those with good/perfect adherence (33.5 months vs 92.2; P = 0.001) and among those with intermediate adherence than among those with good/perfect adherence (22.5 months vs 92.2; P<0.001; Figure 1B). This result was confirmed by multivariable analysis in which confounders such as age and sex were controlled (Table 1).

**Table 1:** Risk factors for first-line HAART discontinuation among antiretroviral-naive paediatric patients.

	Univariate (N = 104)				Multivariable (N = 104)	
	N.	N. of cases (%)	HR (95% CI)	P	HR (95% CI)	P
Age at HAART initiation, years						
≤2	45	24 (53.3)	1		1	
2–12	50	28 (56.0)	0.78 (0.45–1,34)	.36	0.80 (0.45–1,41)	.44
>12	9	5 (55.6)	1.55 (0.59–4.10)	.37	1.38 (0.50–3.81)	.53
Sex						
Girls	58	28 (48.3)	1		1	
Boys	46	29 (63.0)	1.63 (0.97–2.75)	.065	1.59 (0.89–2.83)	.12
Geographical origin						
Spain	73	45 (61.6)	1			
Other	31	12 (38.7)	0.74 (0.39–1.40)	.36		
Adoption						
No	82	45 (54.9)	1			
Yes	22	12 (54.5)	0.79 (0.42–1.49)	.46		
Care						
1 or 2 biological/adoptive parents	80	47 (58.8)	1			
Relatives/Institution	20	10 (50.0)	0.97 (0.49–1.92)	.92		
Unknown	4	–	n.a.			
HIV-1 subtype						
B	53	42 (79.2)	1		1	
Other	20	5 (25.0)	0.41 (0.16–1.04)	.062	0.71 (0.25–2.04)	.53
Unknown	31	10 (32.3)	0.39 (0.19–0.77)	**.** ***007***	0.52 (0.24–1.13)	.10
Baseline HIV-1 RNA log_10_, copies/mL	104	57 (54.8)	1.11 (0.76–1.64)	.58		
Baseline CD4 count^a^, cells/µL	104	57 (54.8)	0.98 (0.95–1.01)	.22		
Clinical status						
A/B	86	46 (53.5)	1			
C	18	11 (61.1)	0.89 (0.46–1.73)	.74		
PMTCT						
No	80	47 (58.8)	1			
Yes	24	10 (41.7)	0.66 (0.33–1.31)	.23		
Breastfeeding						
No	61	34 (55.7)	1			
Yes	27	19 (70.4)	1.62 (0.92–2.87)	.10		
Unknown	16	4 (25.0)	0.60 (0.21–1.70)	.34		
Year of HAART initiation						
>2001	46	17 (37.0)	1		1	
≤2001	47	40 (85.1)	1.97 (1.11–3.50)	**.** ***021***	1.34 (0.68–2.63)	.40
First-line regimen^b^						
PI-based	77	42 (54.5)	1			
NNRTI-based	26	14 (53.8)	1.33 (0.72–2.46)	.36		
Adherence						
Good/perfect	67	29 (43.3)	1		1	
Poor/intermediate	37	28 (75.7)	3.42 (2.01–5.84)	***<.001***	2.60 (1.44–4.70)	**.** ***002***

Legend: HR, hazard ratio; CI, confidence intervals; PMTCT, antiretroviral prophylaxis of HIV mother-to-child transmission; NNRTI, non-nucleoside reverse transcriptase inhibitor; PI, protease inhibitor; n.a., not available; ^a^per 100 cells/µL; ^b^excludes one patient who was treated with a triple NNRTI.

## Discussion

The availability of HAART has transformed the HIV-1 infection into a chronic and treatable disease. As perinatally-infected children likely require life-long treatment, the assessment of the duration of initial and successive HAART regimens is crucial. This study evaluated the duration of initial and successive HAART regimens and the predictors of therapy discontinuation in HIV-1-infected children of CoRISpeS-Madrid Cohort from 1996 up to 2012. This cohort is highly representative of the paediatric HIV-1 community throughout the *Comunidad Autónoma de Madrid* (Spain) as it includes all the cases of vertical HIV-1 transmission that occurred in this area since the dawn of the epidemic in 1982 [30]. Among 104 subjects with median follow-up of 8 years, the 54% discontinued initial HAART with a cumulative incidence of 16% and 38% by 1 and 3-year, respectively. Along with a sustained immunovirological response, we observed a long duration of initial HAART (65 months). The primary reason for first-line regimen discontinuation was poor adherence to antiretrovirals. This is in accordance with the results of the multivable analysis, which indicated more than 2-fold higher likelihood of discontinuation among individuals with non-adherence than those with good/perfect adherence. No association was observed between first-line discontinuation and baseline CD4 count, VL or type of regimen.

Information on the duration of HAART in the paediatric population worldwide is still scarce. High rates of initial HAART change (78%) and an initial HAART duration of only 40 months were observed among children attending a Spanish hospital [31]. In contrast, a large observational study of European children reported that 65% remained on first-line antiretrovirals without treatment interruption after 5 years [32]. Our results are between these two studies with 55% of the children remaining on first-line antiretrovirals without treatment interruption after 5 years. Difference in the definition of regimen discontinuation and in the characteristics of the study population, that included either therapy-experienced or therapy-naïve children, may account for the longer initial HAART duration observed in our study. Despite these differences, non-adherence to treatment was the main determinant of first regimen duration like in our study [31]. As in these previous studies, poor adherence to antiretroviral treatment was the main determinant of the first regimen duration in our children indicating that measures to increase the levels of adherence to therapy in this population are required to extend the duration of initial HAART regimen.

Additional information for the clinical management of HIV-1-infected children and adolescents derives from a limited number of studies that included HIV-1-infected adults. Some observational surveys corroborate our results, showing high rates of initial regimen discontinuation [33]–[35]. Concerning the duration of the initial HAART regimen in HIV-infected adults, Chen R.Y. *et al*. reported a median duration of only 1.6 years, while the successive regimens were considerably shorter [36]. A study that included both treatment-experienced and treatment-naïve patients reported a similar duration of almost one year and durability was associated with being pre-HAART therapy naïve, prompt response to HAART and PI-based initial regimen [37]. Antiretrovirals-associated toxicity was described as important determinants of discontinuation and short duration of the therapy [33], [34], [36].

Missing baseline data were a limitation in this study, as did not allow the inclusion of all antiretroviral-naïve children enrolled in the Cohort who initiated HAART. Moreover, data on socio-economic status or specific treatment-related toxicities were available for a subgroup of the study population, thus we were not able to fully capture their effect on therapy discontinuation. Nevertheless, these missing data do not affect the overall HAART duration estimates. Despite the exclusion of some patients from this survey might represent a potential source of bias, it is of note that several characteristics of included and not included patients were comparable. Another limitation of this study is that adherence was not included in the analyses as a time-dependent variable. Notwithstanding these limitations, the long duration of initial HAART represents a relevant and encouraging finding, indicating that the likelihood that effective HAART will last a lifetime is possible. However, enthusiasm for these findings may be dampened by concerns for the risk of suboptimal adherence observed in those patients who might benefit from additional adherence strategies in routine clinical care.

## Annex

Paediatric hospitals of the CoRISpES-Madrid Cohort working group: Hospital Universitario “Doce de Octubre”, Madrid (29 patients); Hospital Universitario “La Paz”, Madrid (14 patients); Hospital Universitario de Getafe, Madrid (13 patients); Hospital Universitario “Carlos III”, Madrid (12 patients); Hospital General Universitario “Gregorio Marañón”, Madrid (12 patients); Hospital Infantil Universitario “Niño Jesús”, Madrid (8 patients); Hospital de Móstoles (7 patients); Hospital Príncipe de Asturias, Alcalá de Henares, Madrid (6 patients); Hospital de Torrejón de Ardoz, Madrid (3 patients).

## Supporting Information

File S1 Supporting Information Table S1, Baseline characteristics and antiretroviral therapy use among the study population. Legend: ^a^median (IQR); ^b^number (%); ^c^median (95%CI); *C (n = 3), G (n = 2), A1 (n = 1), F1 (n = 1); **recombinant subtypes: CRF01_AE (n = 1), CRF02_AG (n = 10), CRF12_BF (n = 1), CRF13_cpx (n = 1); ^#^3TC/ddI+d4T+NFV (n = 23; 48.9%); ^##^AZT/ABV +3TC+KLT (n = 16; 53.3%); ^∧^d4T+ddI+EFV (n = 7; 29.2%); ^∧∧^AZT +3TC+ABV+NVP (n = 2; 100%); ^∧∧∧^AZT +3TC+ddI (n = 1; 100%). CRF, circulating recombinant form; HAART, highly active antiretroviral therapy; MTCT, mother-to-child transmission; PMTCT, antiretroviral prophylaxis of HIV mother-to-child transmission; CMV, cytomegalovirus; HBV, hepatitis B virus; NRTI, nonnucleoside/nucleotide reverse-transcriptase inhibitor; NNRTI, nonnucleoside reverse-transcriptase inhibitor; PI, protease inhibitor. Supporting Information Table S2, Characteristics of included and not included patients. Legend: ^$^Fisher's exact test (2-tailed); ^$$^Mann-Whitney U test (2-tailed). Supporting Information Table S3, Analysis of the cause of discontinuation of first-line HAART according to the type of regimen. Legend: NNRTI, nonnucleoside reverse-transcriptase inhibitor; PI, protease inhibitor; *the underlying cause of death was dideoxynucleoside-induced severe lactic acidosis. Supporting Information Figure S1, Evolution of the percentage of the most frequent antiretroviral drug over time (A) and evolution of viral load (VL), %CD8 and %CD4 over time (B). Legend: Mean of log_10_ VL (copies/mL), CD8^+^ T-cell percentage and CD4^+^ T-cell percentage; bars represent 2 s.e.m. EFV, efavirenz; KLT, kaletra; NFV, nelfinavir.
(DOCX)
