# Supplementary material for: Determinants of Highly Active Antiretroviral Therapy Duration in HIV-1-Infected Children and Adolescents in Madrid, Spain, from 1996 to 2012
Source: PLoS One. 2014 May 1;9(5):e96307. doi: 10.1371/journal.pone.0096307 (PMC4006876; doi:10.1371/journal.pone.0096307)
Supplement: File S1 — Supporting Information Table S1, Baseline characteristics and antiretroviral therapy use among the study population. Legend: amedian (IQR); bnumber (%); cmedian (95%CI); *C (n = 3), G (n = 2), A1 (n = 1), F1 (n = 1); **recombinant subtypes: CRF01_AE (n = 1), CRF02_AG (n = 10), CRF12_BF (n = 1), CRF13_cpx (n = 1); #3TC/ddI+d4T+NFV (n = 23; 48.9%); ##AZT/ABV +3TC+KLT (n = 16; 53.3%); ∧d4T+ddI+EFV (n = 7; 29.2%); ∧∧AZT +3TC+ABV+NVP (n = 2; 100%); ∧∧∧AZT +3TC+ddI (n = 1; 100%). CRF, circulating recombinant form; HAART, highly active antiretroviral therapy; MTCT, mother-to-child transmission; PMTCT, antiretroviral prophylaxis of HIV mother-to-child transmission; CMV, cytomegalovirus; HBV, hepatitis B virus; NRTI, nonnucleoside/nucleotide reverse-transcriptase inhibitor; NNRTI, nonnucleoside reverse-transcriptase inhibitor; PI, protease inhibitor. Supporting Information Table S2, Characteristics of included and not included patients. Legend: $Fisher's exact test (2-tailed); $$Mann-Whitney U test (2-tailed). Supporting Information Table S3, Analysis of the cause of discontinuation of first-line HAART according to the type of regimen. Legend: NNRTI, nonnucleoside reverse-transcriptase inhibitor; PI, protease inhibitor; *the underlying cause of death was dideoxynucleoside-induced severe lactic acidosis. Supporting Information Figure S1, Evolution of the percentage of the most frequent antiretroviral drug over time (A) and evolution of viral load (VL), %CD8 and %CD4 over time (B). Legend: Mean of log10 VL (copies/mL), CD8+ T-cell percentage and CD4+ T-cell percentage; bars represent 2 s.e.m. EFV, efavirenz; KLT, kaletra; NFV, nelfinavir. (DOCX) [file pone.0096307.s001.docx]

**Supporting Information Table 1. Baseline characteristics and antiretroviral therapy use among the study population**

| **Baseline characteristics** |  | **All patients (N=104)** |
| --- | --- | --- |
| Age, years ^a^ |  | 2.6 (0.4-7.5) |
| Age at first regimen start, years ^b^ |  |  |
| ≤2 |  | 45 (43.3) |
| 2-12 |  | 50 (48.1) |
| >12 |  | 9 (8.7) |
| Girls ^b^ |  | 58 (55.8) |
| Spain origin ^b^ |  | 73 (70.2) |
| Care ^b^ |  |  |
| 1 or 2 biological parents |  | 61 (58.7) |
| 1 or 2 adoptive parents |  | 19 (18.3) |
| Relatives |  | 16 (15.4) |
| Residential institution |  | 4 (3.8) |
| Unknown |  | 4 (3.8) |
| HIV-1 subtype ^b^ |  |  |
| B |  | 53 (51.0) |
| Other ^*^ |  | 7 (6.7) |
| Recombinant ^**^ |  | 13 (12.5) |
| Unknown |  | 31 (29.8) |
| HIV-1 RNA, copies/mL ^a^ |  | 114,000 (41,247-391,250) |
| HIV-1 RNA, log_10_ copies/mL ^a^ |  | 5.1 (4.6-5.6) |
| CD4 count, cells/µL ^a^ |  | 769 (276-1,671) |
| %CD4 ^a^ |  | 21.5 (12.3-34.0) |
| CD8 count, cells/µL (N=89) ^a^ |  | 1,270 (906-1,934) |
| %CD8 (N=89) ^a^ |  | 39.0 (27.3-54.0) |
| Immune category ^b^ |  |  |
| ≥500 cells/µL |  | 66 (63.5) |
| 200-499 cells/µL |  | 19 (18.3) |
| <200 cells/µL |  | 19 (18.3) |
| AIDS status ^b^ |  | 18 (17.3) |
| MTCT of co-infections ^b^ |  |  |
| CMV |  | 7 (6.7) |
| HBV |  | 1 (1.0) |
| PMTCT |  |  |
| No |  | 80 (76.9) |
| Yes |  | 24 (23.1) |
| Breastfeeding |  |  |
| No |  | 61 (58.7) |
| Yes |  | 27 (26.0) |
| Unknown |  | 16 (15.4) |
| **Antiretroviral therapy** |  |  |
| Switch of regimen ^b^ |  |  |
| One switch |  | 35 (65.5) |
| Two switches |  | 12 (21.4) |
| Three to seven switches |  | 9 (16.1) |
| HAART duration (month) ^c^ |  |  |
| First-line regimen |  | 64.5 (47.5-81.5) |
| Second-line regimen |  | 69.8 (9.9-129.6) |
| Third-line regimen |  | 66.5 (38.0-94.9) |
| Type of first-line regimen ^b^ |  |  |
| 2 NRTI + 1 PI ^#^ |  | 47 (45.2) |
| 2 NRTI + 1 boosted PI ^##^ |  | 30 (28.8) |
| 2 NRTI + 1 NNRTI ^^^ |  | 24 (23.1) |
| 3 NRTI + 1 NNRTI ^^^^ |  | 2 (1.9) |
| 3 NRTI ^^^^^ |  | 1 (1.0) |
| Type of second-line regimen ^b^ |  |  |
| 2 NRTI + 1 PI |  | 6 (10.7) |
| 2 NRTI + 1 boosted PI |  | 20 (35.7) |
| 2 NRTI + 1 NNRTI |  | 16 (28.6) |
| 3 NRTI |  | 4 (7.1) |
| Other (3-4 drugs) |  | 10 (17.9) |
| Type of third-line regimen ^b^ |  |  |
| 2 NRTI + 1 PI |  | -- |
| 2 NRTI + 1 boosted PI |  | 8 (38.1) |
| 2 NRTI + 1 NNRTI |  | 6 (28.6) |
| 3 NRTI |  | 1 (4.8) |
| Other (4-6 drugs) |  | 6 (28.6) |
|  |  |  |
|  |  |  |
|  |  |  |
|  |  |  |
|  |  |  |
|  |  |  |
|  |  |  |
|  |  |  |
|  |  |  |

Legend: ^a^ median (IQR); ^b^ number (%); ^c^ median (95%CI); ^*^ C (n=3), G (n=2), A1 (n=1), F1 (n=1); ^**^ recombinant subtypes: CRF01_AE (n=1), CRF02_AG (n=10), CRF12_BF (n=1), CRF13_cpx (n=1); ^#^ 3TC/ddI + d4T + NFV (n=23 ; 48.9%); ^##^ AZT/ABV + 3TC + KLT (n= 16; 53.3%); ^^^ d4T + ddI + EFV (n=7; 29.2%); ^^^^ AZT + 3TC + ABV + NVP (n=2; 100%); ^^^^^ AZT + 3TC + ddI (n=1; 100%). CRF, circulating recombinant form; HAART, highly active antiretroviral therapy; MTCT, mother-to-child transmission; PMTCT, antiretroviral prophylaxis of HIV mother-to-child transmission; CMV, cytomegalovirus; HBV, hepatitis B virus; NRTI, nonnucleoside/nucleotide reverse-transcriptase inhibitor; NNRTI, nonnucleoside reverse-transcriptase inhibitor; PI, protease inhibitor.

**Supporting Information Table 2. Characteristics of included and not included patients**

| **Characteristics** | **Patients included** | **Patients not included** | **P** |
| --- | --- | --- | --- |
| Sex | **N = 104** | **N = 20** |  |
| Girls | 58 (85.3) | 10 (14.7) | .81^$^ |
| Boys | 46 (82.1) | 10 (17.9) |  |
| Origin | **N = 104** | **N = 20** |  |
| Spain | 73 (85.9) | 12 (14.1) | .43^$^ |
| Other | 31 (79.5) | 8 (20.5) |  |
| Clinical status at HAART initiation | **N = 104** | **N = 17** |  |
| No-C | 86 (85.1) | 15 (14.9) | .74^$^ |
| C | 18 (90.0) | 2 (10.0) |  |
| Breastfeeding | **N = 88** | **N = 16** |  |
| No | 61 (80.3) | 15 (19.7) | .06 |
| Yes | 27 (96.4) | 1 (3.6) |  |
| Maternal transmission category | **N = 79** | **N = 12** |  |
| Injection drug use | 41 (80.4) | 10 (19.6) | .60^$^ |
| Heterosexual | 38 (95.0) | 2 (5.0) |  |
| Age at HAART initiation | **N = 104** | **N = 18** | .12^$$^ |
| Median, yrs | 2.6 (0.4-7.5) | 1.3 (0.3-3.7) |  |

Legend: ^$^Fisher's exact test (2-tailed); ^$$^Mann-Whitney U test (2-tailed).

**Supporting Information Table 3. Analysis of the cause of discontinuation of first-line HAART according to the type of regimen**

| **First-line HAART** | | | | |
| --- | --- | --- | --- | --- |
|  | **Total**  N (%) | **PI-based**  N (%) | **NNRTI-based** N (%) | **Other**  N (%) |
| Tot. cases of discontinuation | 57 (100.0) | 42 (100.0) | 14 (100.0) | 1 (100.0) |
| Treatment failure | 19 (33.3) | 17 (40.5) | 2 (14.3) | -- |
| Poor adherence | 12 (21.1) | 8 (19.0) | 3 (21.4) | 1 (100.0) |
| Simplification | 10 (17.5) | 7 (16.7) | 3 (21.4) | -- |
| Toxicity | 6 (10.5) | 4 (9.5) | 2 (14.3) | -- |
| Social/family problem | 4 (7.0) | 3 (7.1) | 1 (7.1) | -- |
| Decision of patient/parents/tutor | 3 (5.3) | 1 (2.4) | 2 (14.3) | -- |
| Planned treatment interruption | 2 (3.5) | 2 (4.8) | -- | -- |
| Death* | 1 (1.8) | -- | 1 (7.1) | -- |

Legend: NNRTI, nonnucleoside reverse-transcriptase inhibitor; PI, protease inhibitor; * the underlying cause of death was dideoxynucleoside-induced severe lactic acidosis.

**Supporting Information Figure 1. A) Evolution of the percentage of the most frequent antiretroviral drug over time. B) Evolution of viral load (VL), %CD8 and %CD4 over time**

| **A** | **B** |
| --- | --- |
| **** | **** |

Legend: Mean of log_10_ VL (copies/mL), CD8^+^ T-cell percentage and CD4^+^ T-cell percentage; bars represent 2 s.e.m. EFV, efavirenz; KLT, kaletra; NFV, nelfinavir.
